# Supplementary material for: Acute Stress Effects over Time on Gene Expression Patterns in the Male Zebrafish (Danio rerio) Brain
Source: Animals (Basel). 2025 Aug 19;15(16):2431. doi: 10.3390/ani15162431 (PMC12382661; doi:10.3390/ani15162431)
Supplement: Supplementary file 1 [file animals-15-02431-s001.zip › Supplement Males V2.pdf]

Supplement

Figure S1. Gene expression profiles (expressed as marginal means derived from the Bayes models performed on the log2 values of the normalized gene expression values) in the telencephalon of male control fish (group 1) and fish after the treatment (30 min = 2; 60 min = 3; 90 min = 4) whereby the following different treatments have been applied as described in the Material & Methods section: feed rewarding, feed control, chasing, confinement and air exposure, marginal means ± SEM; n = 6 per treatment, means of groups with the same letters are not significantly different from each other, $p < 0.05$).

3A : Feed Reward

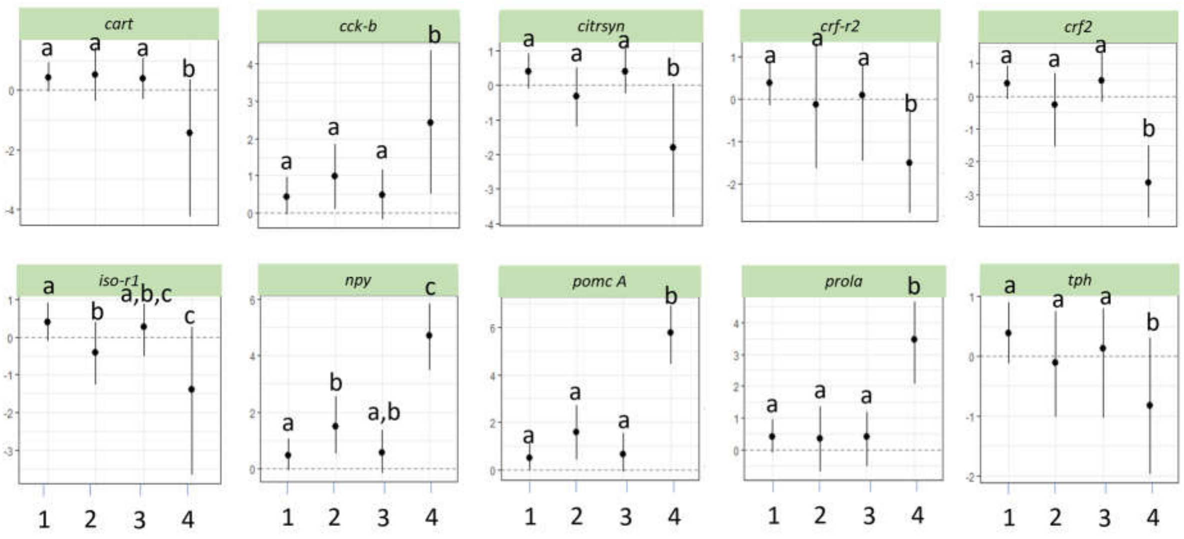

3B : Feed Contr

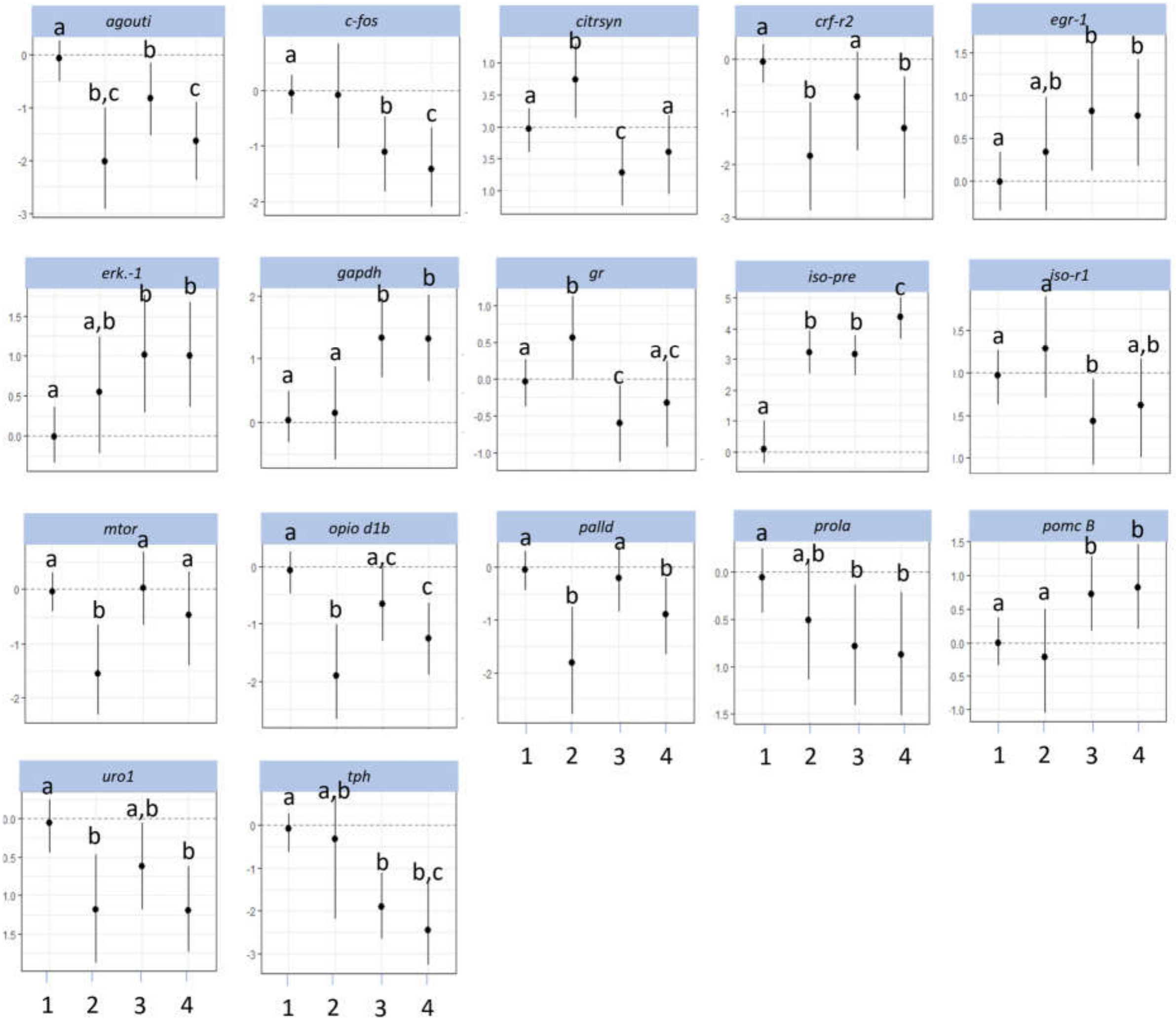

3C: Chasing

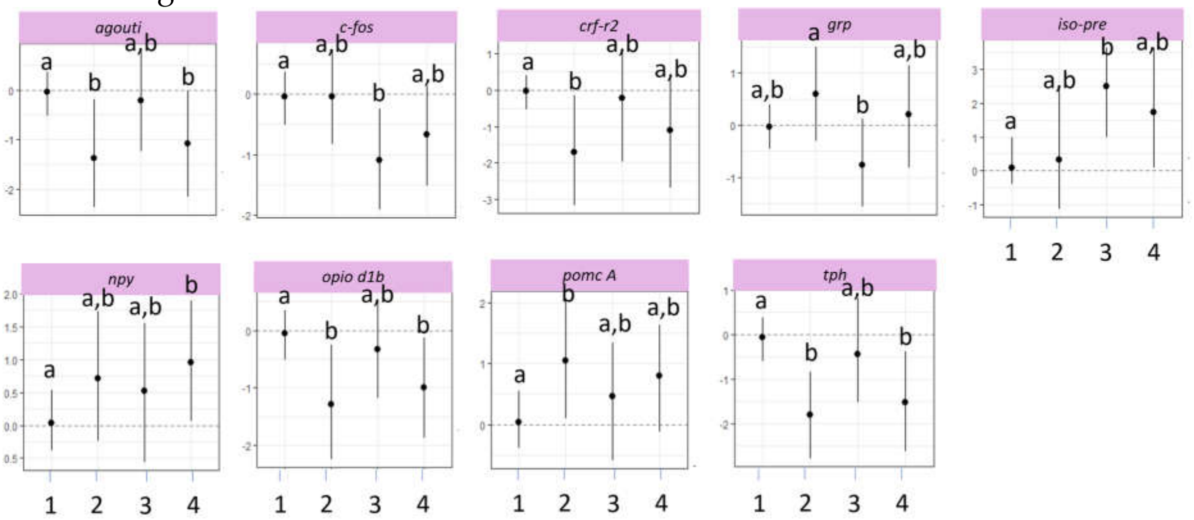

3D : Confinement

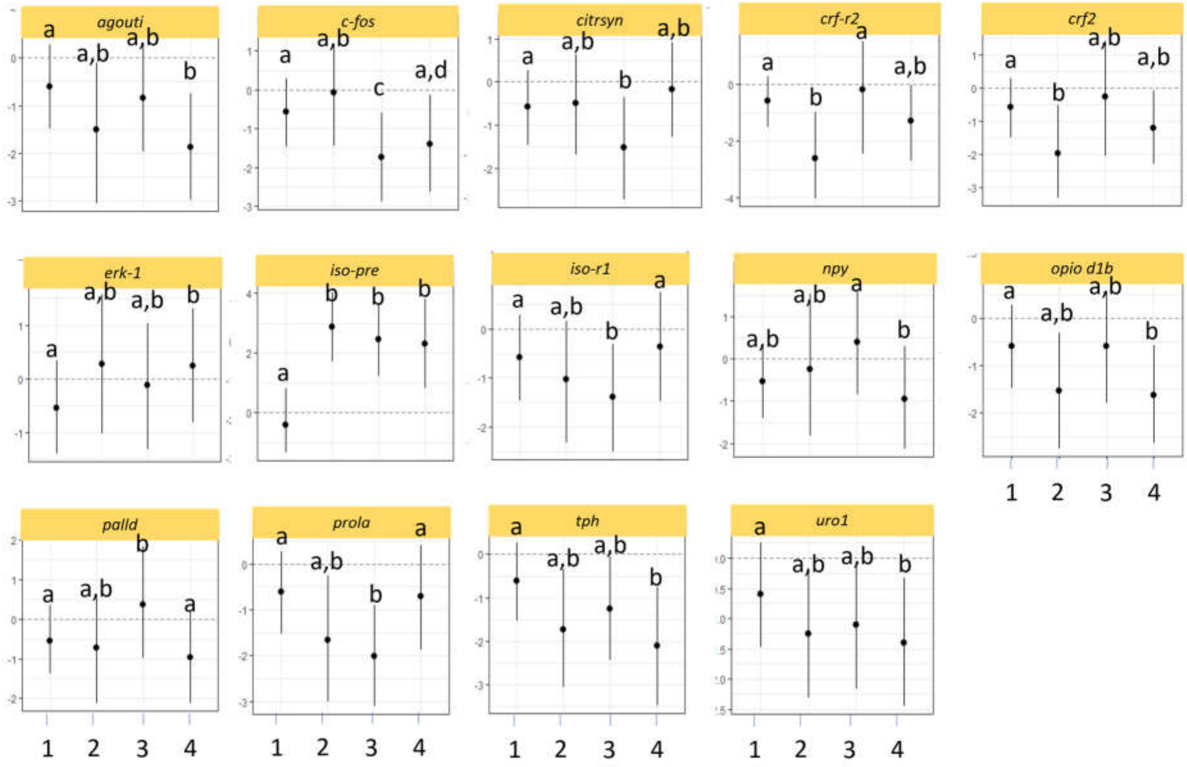

3E:Air

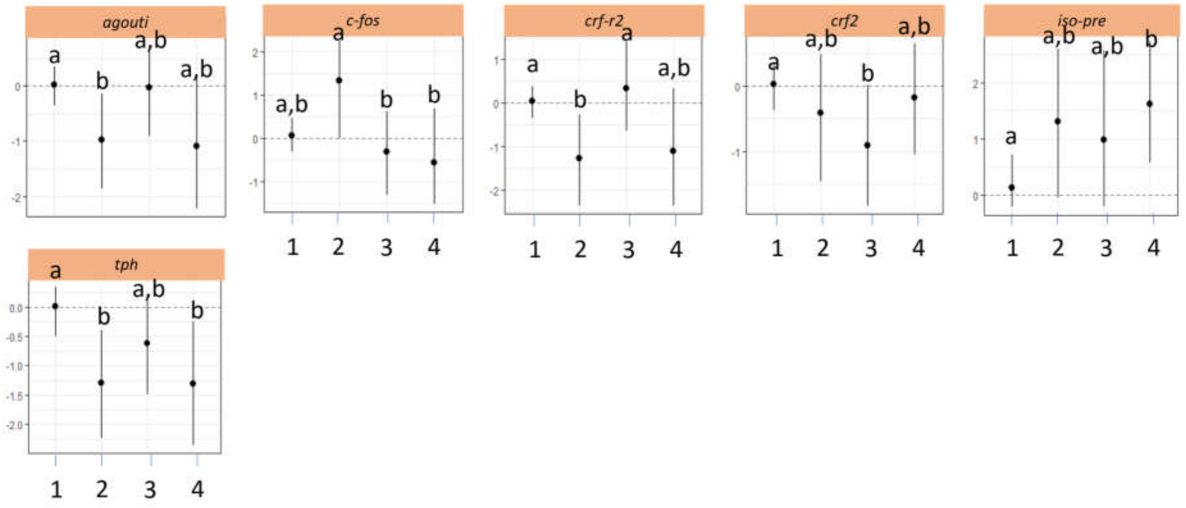

For males the feed rewarding showed no effect on the expression of immediate early genes (IEGs) in the telencephalon (Fig. S3A). However, the expression of the metabolic gene *citrsyn* in male feed control fish was lower in the 90 min group compared with the controls ($p = 0.022$), and significantly different between the fish 60 min and 90 min after treatment ($p = 0.018$). The feed control groups showed significantly higher expression of *erg-1* and *erk-1* and significant lower *c-fos* expression in the 60 min and 90 min group compared with the controls ($p < 0.020$; Fig. S3B). In addition, the expression of *c-fos* was different in the 30 min group compared with the 90 min group ($p = 0.004$), whereas the *palld* expression was significantly lower 30 min and 90 min after treatment compared with the controls ($p \leq 0.024$). In the feed control fish, also the expression of *citrsyn* was significantly higher 30 min after treatment and lower 60 min after treatment compared with the controls ($p \leq 0.018$). In addition, the

expression of *gapdh* was significantly higher 60 min and 90 min after the treatment compared with the remaining the fish 30 min after treatment and the controls ($p \leq 0.042$). The expression of *c-fos* was also significantly lower in the 60 min group of the chased animals compared with the controls ($p = 0.008$; Fig. S3C). The *c-fos* expression was also found to be lower in the confined individuals at 60 min after treatment compared with the controls ($p = 0.014$; Fig. S3D). The expression of *palld* was significantly higher in confined animals 60 min after treatment compared with the fish sampled 90 min after the treatment ($p = 0.048$). Confinement also resulted in significantly lower *citrsyn* expression 60 min after treatment compared with the controls ($p = 0.040$), and also the difference between the 60 min group and the 90 min group was significantly different ($p = 0.016$). Air exposure resulted in a lower expression of *c-fos* of males sampled 60 min and 90 min after treatment compared with the 30 min group ($p \leq 0.046$; Fig. S3E).

For feed-rewarded males, the telencephalon showed mainly effects on HPI axis-related genes 90 min after treatment compared with the other treatments (Fig. S3A). Accordingly, the expression of *crf-2* and *crf-r2* was significantly lower ($p \leq 0.006$) in the 90 min group than in the remaining groups. However, there was a significantly lower *crf2* expression in the 90 min group than in the remaining treatment time points ($p \leq 0.002$). In fish belonging to the feed control groups, the expression of *crf-r2* was significantly lower in the 30 min and 90 min group than in the controls ($p \leq 0.014$; Fig. S3B). The same fish showed a significantly higher *gr* expression 30 min after treatment and a lower *gr* expression 60 min after treatment compared with the controls ($p \leq 0.046$), whereby the difference between the 30 min treatment and the 60 min and 90 min treatments was also significant ($p \leq 0.014$). Moreover, the feed control fish showed a significantly lower *uro1* expression 30 min and 90 min after treatment compared with the controls ($p \leq 0.002$). The expression of *pomc B* was significantly higher 60 min and 90 min after feed control treatment compared with the fish sampled 30 min after the treatment and the controls ($p \leq 0.044$). Chasing resulted in significantly lower *crf-r2* and higher *pomc A* expression in the fish 30 min after treatment compared with the controls ($p \leq 0.036$; Fig. S3C). Confinement led to lower *crf2* and *crf-r2* expression in fish 30 min after treatment compared with the controls ($p \leq 0.022$; Fig. S3D). For *crf-r2* the difference between the 30 min group and the 60 min group was also significant ($p = 0.032$). Finally, confinement resulted in significantly lower *uro1* expression in fish 90 min after treatment compared with the controls ($p = 0.032$). In air-exposed fish, a significantly lower *crf2* expression was observed compared with the controls ($p \leq 0.040$; Fig. S3E).

For feed-rewarded females, the telencephalon showed mainly effects on appetite-related gene 90 min after treatment compared with the other time points (Fig. S3A). Accordingly, the expression of *npy* was significantly higher ($p < 0.001$), but the *cart* expression was found to be significantly lower in the 90 min group compared with the remaining treatment groups ($p \leq 0.044$). In addition, a significantly higher *npy* expression was observed in the 30 min group compared with the controls ($p = 0.026$). Furthermore, the expression of *cck-b* was significantly higher in the 90 min group compared with the controls ($p = 0.040$). The expression of *agouti* in the feed control animals was significantly lower than in the controls ($p \leq 0.036$; Fig. S3B), and also lower in the fish 90 min after treatment compared with the animals 60 min after treatment ($p = 0.026$). Chasing resulted in lower *grp* expression compared with the fish 30 min after treatment ($p = 0.026$; Fig. S3C), but higher expression of *npy* in the fish 90 min after treatment compared with the controls ($p = 0.030$). In addition, chased fish showed significantly decreased *agouti* expression in fish 30 min and 90 min after treatment ($p \leq 0.042$). Confinement resulted in a significantly lower *npy* expression 90 min after treatment compared with the 60 min group ($p = 0.032$; Fig. S3D). Furthermore, the expression of *agouti* was significantly lower in the 90 min group compared with the controls ($p = 0.004$). Air exposure resulted in lower *agouti* expression 30 min after treatment compared with the controls ($p = 0.020$; Fig. S3E).

Furthermore, the expression of *tph* and *iso-r1* in the telencephalom was significantly lower in the 90 min group than in the controls ($p \leq 0.042$) of feed-rewarded animals (Fig. S3A). However, there was a significantly lower *prola* expression in the 90 min group than in the remaining treatment groups ($p < 0.001$). Finally, the *iso-r1* in feed-rewarded animals was significantly lower in the 30 min group than in the controls ($p = 0.036$). Fish belonging to the feed control group showed lower *prola* expression in the 60 min and 90 min group than in the controls ($p \leq 0.032$; Fig. S3B). The expression of *iso pre* in animals of these treatment groups was higher than in the controls ($p < 0.001$), and also higher 90 min after the treatment compared with the fish sampled 30 min and 60 min after treatment ($p \leq 0.016$). The expression of *iso-r1* was found to be lower in animals 60 min after the treatment compared with the controls ($p = 0.048$). Moreover, the expression of *mtor* was significantly lower 30 min after the treatment compared with the remaining treatment groups ($p \leq 0.042$). Finally, the *tph* expression was influenced by the feed control treatment with significantly lower expression 60 min and 90 min after treatment compared with the controls ($p < 0.001$), whereas the expression of the *opio d1b* was significantly lower in fish 30 min and 90 min after the treatment compared with the controls ($p < 0.001$) and different between fish 30 min and 60 min after the treatment ($p = 0.024$). Chasing resulted in higher expression of *iso pre* in fish 60 min after treatment compared with the controls ($p = 0.002$; Fig. S3C). In addition, chased fish showed lower *tph* and *opio d1b* expression 30 min and 90 min after treatment compared with the controls ($p < 0.026$). Moreover, confinement led to lower *prola* expression in fish 60 min after treatment compared with the controls ($p = 0.002$; Fig. S3D), and a significant difference also between the fish 60 min and 90 min after treatment ($p = 0.024$). Confined animals also showed significantly higher *iso pre* expression compared with the controls ($p = 0.024$). Finally, confinement resulted in significantly lower *tph* and *opi d1b* expression in fish 90 min after treatment compared with the controls ($p = 0.032$), and the expression of *iso-r1* was significantly lower in fish 60 min after treatment compared with the controls and the fish 90 min after treatment ($p \leq 0.046$). Air exposure influenced the *iso pre* expression which was higher in the fish 90 min after treatment compared with the controls ($p = 0.006$; Fig. S3E). In air-exposed fish also the expression of *tph* was lower 30 min and 90 min after treatment compared with the controls ($p \leq 0.014$).

Figure S2. Gene expression profiles (expressed as marginal means derived from the Bayes models performed on the log2 values of the normalized gene expression values) in the hypothalamus of male control fish (group 1) and fish after the treatment (30 min = 2; 60 min = 3; 90 min = 4) whereby the following different treatments have been applied as described in the Material & Methods section: feed rewarding, feed control, chasing, confinement and air exposure, marginal means ± SEM; n = 6 per treatment, means of groups with the same letters are not significantly different from each other, $p < 0.05$).

4A : Feed reward

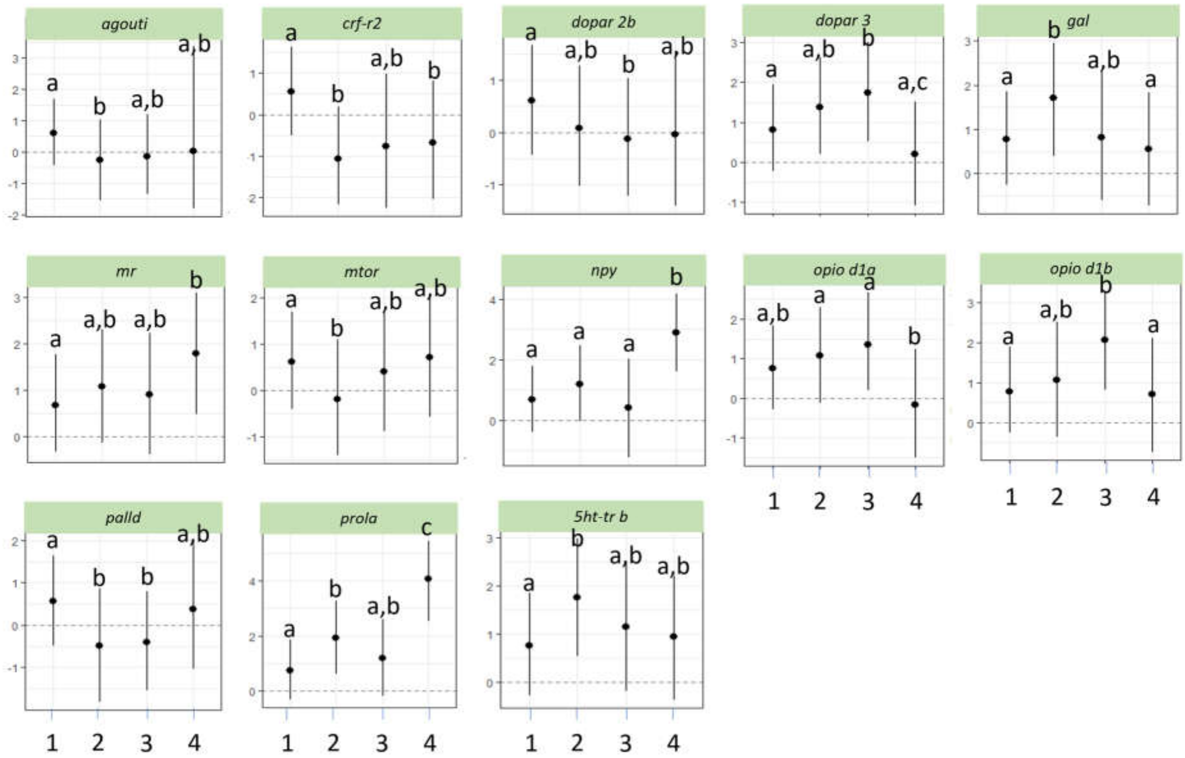

4B : Feed Contr

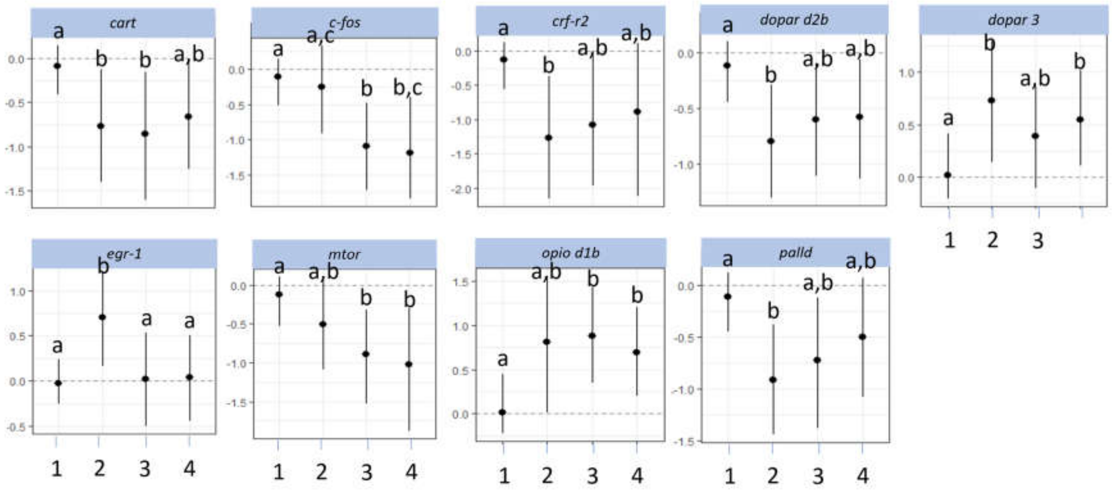

4C : Chasing

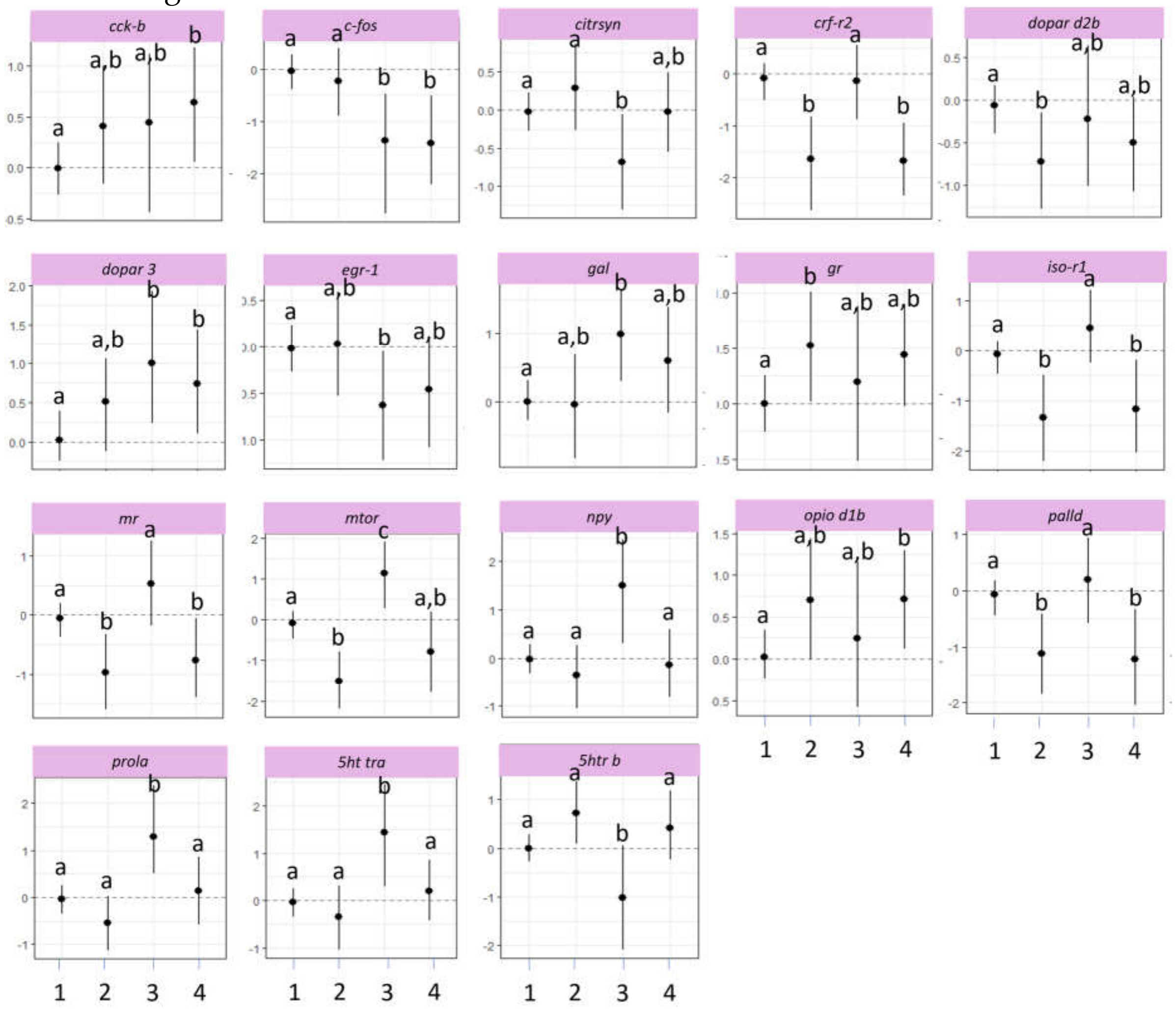

4D : Confinement

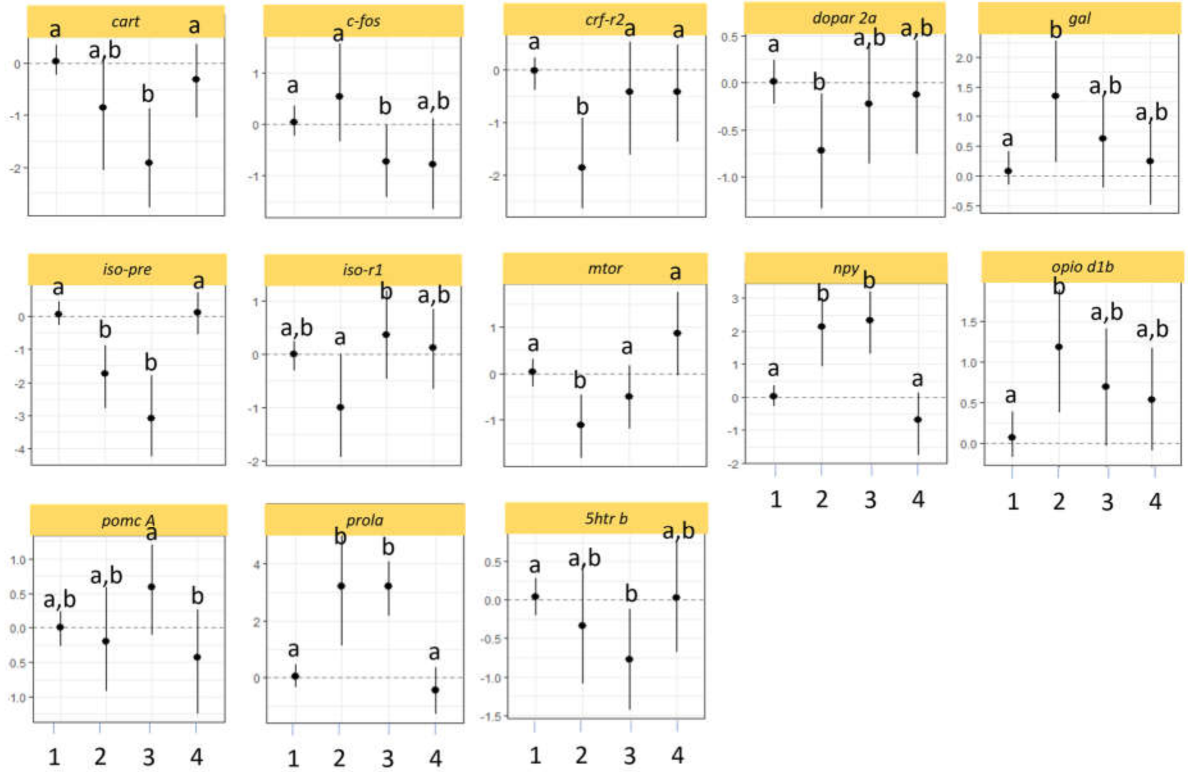

4E:Air

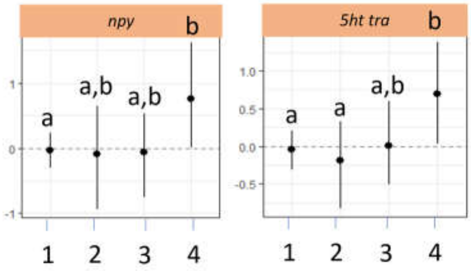

For the hypothalamus of males, the feed rewarding had a reducing effect on the *palld* expression 30 min and 60 min after the treatment compared with the controls ($p \leq 0.006$; Fig. S4A). In addition, the expression of *egr-1* was higher and the expression of *palld* significantly lower after the feed control treatment 30 min after treatment compared with the remaining treatments ($p \leq 0.048$; Fig. S4B). In the same fish, the expression of *c-fos* was lower in the 60 min and 90 min group compared with the controls and the fish sampled 30 min after the treatment ($p \leq 0.044$). In the chased fish, the expression of *egr-1* in the hypothalamus was significantly lower in the fish 60 min after the treatment compared with the controls ($p = 0.046$; Fig. S4C), while the expression of *c-fos* was lower 60 min and 90 min after treatment compared with the fish sampled 30 min after treatment and the controls ($p \leq 0.010$). Moreover, the expression of *palld* in chased animals was found to be lower 30 min and 90 min after treatment compared with the controls ($p \leq 0.014$). In the same fish, the expression of *citrsyn* significantly lower in fish 60 min after the treatment compared with the controls and fish sampled 30 min after treatment ($p \leq 0.042$). In confined fish, the expression of *c-fos* in the hypothalamus was significantly lower in the fish 60 min after the treatment compared with the controls and fish sampled 30 min after treatment ($p \leq 0.044$; Fig. S4D). In the hypothalamus of air-exposed fish, no significant differences in the expression of immediate early genes were observed (data not shown).

For the hypothalamus of males, the expression of *crf-r2* was significantly lower in the 30 min and in the 90 min group than in the controls ($p \leq 0.046$; Fig. S4A). In contrast, the expression of *mr* was significantly higher in the 90 min group than in the controls ($p = 0.018$). In the feed control fish, the expression of *crf-r2* in the hypothalamus was significantly lower in the fish 30 min after the treatment compared with the controls ($p = 0.012$; Fig. S4B). In the chased fish, the expression of *crf-r2* was significantly different 30 min and 90 min after the treatment compared with the controls ($p < 0.001$; Fig. 2B), whereas the expression of *gr* was higher in the 30 min group compared with the controls ($p = 0.030$). In contrast, the expression of *mr* was lower in fish 30 min and 90 min after chasing compared with the controls ($p \leq 0.006$). In the hypothalamus of confined fish, the expression of *crf-r2* was significantly lower in the fish 30 min after the treatment compared with the remaining sampling time points ($p \leq 0.040$; Fig. S4D). The same fish also showed higher *pomc A* expression 60 min after confinement compared with the fish 90 min after treatment ($p = 0.046$). In the hypothalamus of air-exposed fish, no significant differences in the expression of HPI axis-related genes were observed (data not shown).

For the hypothalamus of males, the feed rewarding had an increasing effect on the *npy* expression 90 min after the treatment compared with the remaining treatments ($p \leq 0.006$; Fig. S4A). In the same animals, the expression of *gal* in the hypothalamus was significantly higher in the fish 30 min after the treatment compared with the controls and fish 90 min after the treatment ($p \leq 0.048$). In addition, the *agouti* expression was lower in fish 30 min after the feed control treatment compared with the controls ($p = 0.028$; Fig. S4B). In the feed control fish, the expression of *cart* was significantly lower 30 min and 60 min after the treatment compared with the controls ($p \leq 0.048$). In chased fish, the expression of *npy* was higher in fish sampled 60 min after treatment compared with the remaining treatments ($p \leq 0.010$; Fig. S4C), whereas the expression of *gal* was higher in the 60 min group only compared with the controls

(p = 0.010). In feed control fish, also the expression of *cck-b* was higher in fish sampled 90 min after the treatment compared with the controls (p = 0.026). In the hypothalamus of confined fish, the expression of *cart* was significantly lower 60 min after the treatment compared with the controls and fish sampled 90 min after the treatment (p ≤ 0.012; Fig. S4D). In the same fish, the expression of *npy* was significantly higher 30 min and 60 min after the treatment compared with the remaining groups (p < 0.001). The expression of gal was significantly higher in chased fish 30 min after treatment compared with the controls (p = 0.026) In the hypothalamus of air-exposed fish, the expression of *npy* was significantly higher in fish 90 min after the treatment compared with controls (p = 0.046; Fig. S4E).

For the hypothalamus of males, the feed-rewarded groups showed a significantly higher *prola* expression 30 min and 90 min after the treatment compared with the controls (p < 0.008; Fig. S4A). Moreover, the expression of *dopar 2b* was significantly lower and the expression of *opio d1b* significantly higher in the feed-rewarded fish 60 min after treatment compared with the controls (p ≤ 0.014). In addition, in these animals the expression of *5htrb* was higher and the expression of *mtor* lower 30 min after the treatment compared with the controls (p ≤ 0.024). Finally, the expression of *opio d1a* was significantly lower in the feed-rewarded fish 90 min after the treatment compared with the 60 min and the 30 min treatment group (p ≤ 0.028), and the expression of *dopar 3* was significantly higher in fish sampled 60 min after the treatment compared with the controls. In the feed control fish, the expression of *dopar 2b* in the hypothalamus was significantly lower in the fish 30 min after the treatment compared with the controls (p = 0.012; Fig. S4B). In the same fish, the expression of *mtor* was significantly lower and the *opio d1b* significantly higher 60 min and 90 min after the treatment compared with the controls (p ≤ 0.022). The expression of *dopar3* was significantly higher 30 min and 90 min after the treatment compared with the controls (p ≤ 0.040). In the feed control fish, the expression of *dopar 2b* in the hypothalamus was significantly lower in the fish 30 min after the treatment compared with the controls (p = 0.026; Fig. S4B), and the expression of *5ht-tra* is higher 60 min after the treatment compared with the remaining treatments (p ≤ 0.038). The expression of *5htr b* was significantly lower 60 min after chasing compared with the remaining treatments (p ≤ 0.026; Fig. S4C). In the same fish, the expression of *mtor* was significantly lower in fish 30 min after the treatment and higher in the 60 min group compared with the controls (p ≤ 0.006). In the same treatment groups, the expression of the *opio d1b* was significantly higher in the 90 min group compared with the controls (p = 0.026). The expression of *iso-r1* was significantly lower 30 min and 90 min after the feed control treatment compared with the controls (p ≤ 0.030), whereas the expression of *dopar 3* was significantly higher in fish 60 min and 90 min after treatment (p ≤ 0.034). In the hypothalamus of confined fish, the expression of *prola* was significantly higher in the fish 30 min and 60 min after the treatment compared with the remaining sampling time points (p < 0.001; Fig. S4D). The same fish also showed lower *iso pre* expression 30 and 60 min after confinement compared with the fish 90 min after treatment and the controls (p ≤ 0.002). The expression of *iso-r1* was significantly different between the 30 min and the 60 min treatment (p = 0.016). Confined fish also showed a lower expression of *5htr b* compared with the controls (p = 0.020), and a lower *mtor* expression 30 min after treatment compared with all remaining treatments (p ≤ 0.018). The *dopar 2a* expression in confined fish also showed lower values than in the controls (p = 0.024), while the *opio d1b* expression showed higher expression in the same fish (p = 0.008). In the hypothalamus of air-exposed fish, significant differences in the expression of *5ht tra* were observed between the fish 90 min after treatment and the controls and the 30 min group (p ≤ 0.032; Fig. S4E).

Figure S3. Gene expression profiles (expressed as marginal means derived from the Bayes models performed on the log2 values of the normalized gene expression values) in the optic tectum of male control fish (group 1) and fish after the treatment (30 min = 2; 60 min = 3; 90 min = 4) whereby the following different treatments have been applied as described in the Material & Methods section: feed rewarding, feed control, chasing, confinement and air exposure, marginal means ± SEM; n = 6 per treatment, means of groups with the same letters are not significantly different from each other, $p < 0.05$).

5A : Feed reward

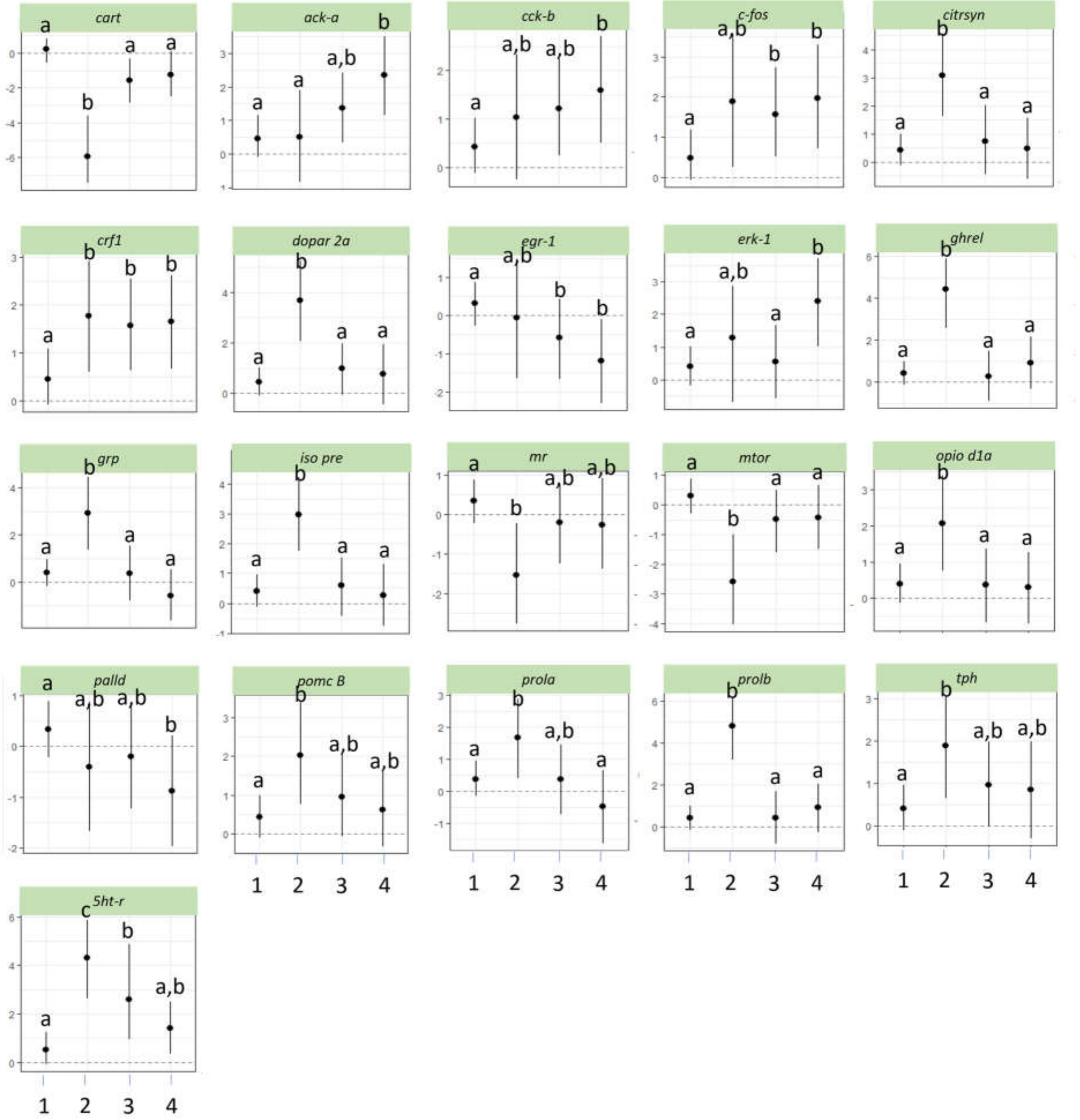

5B : Feed Contr

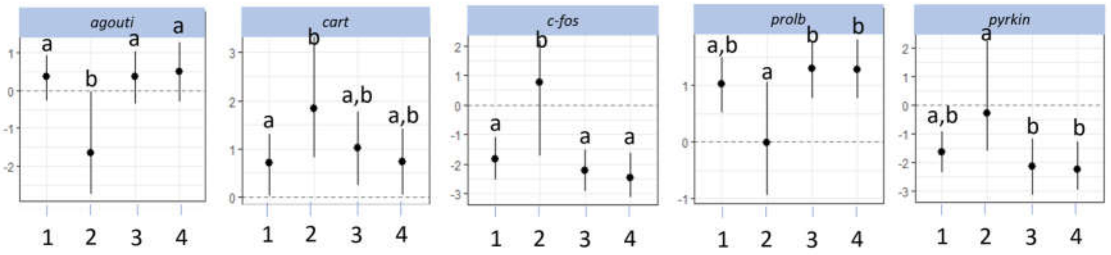

5C : Chasing

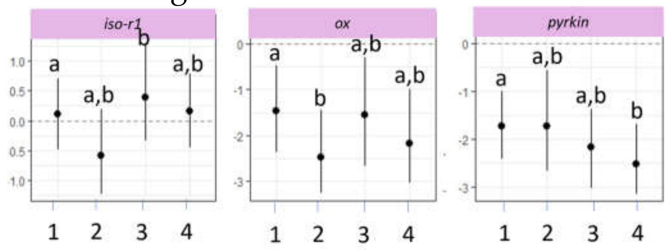

5D : Confinement

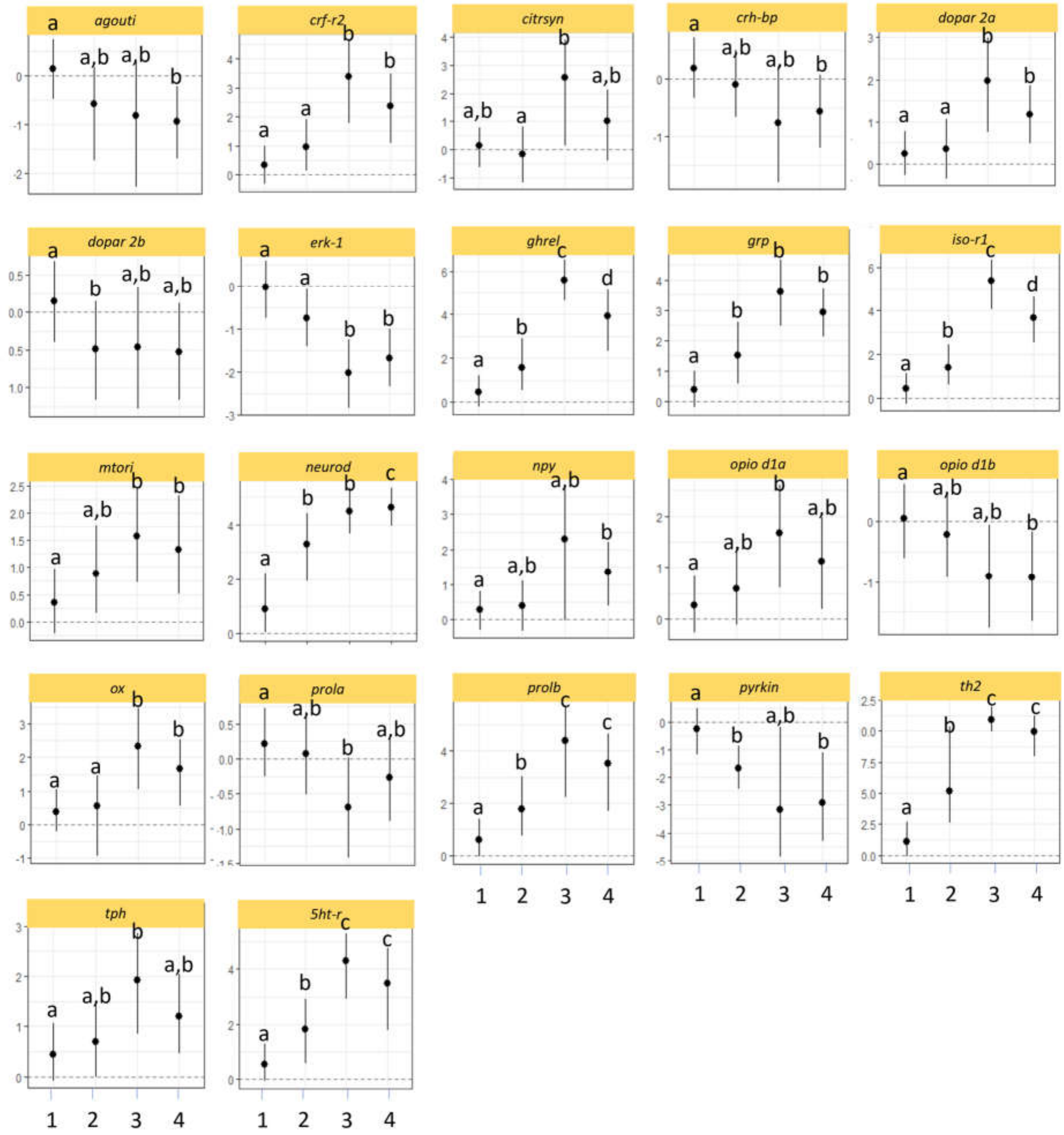

5E : Air

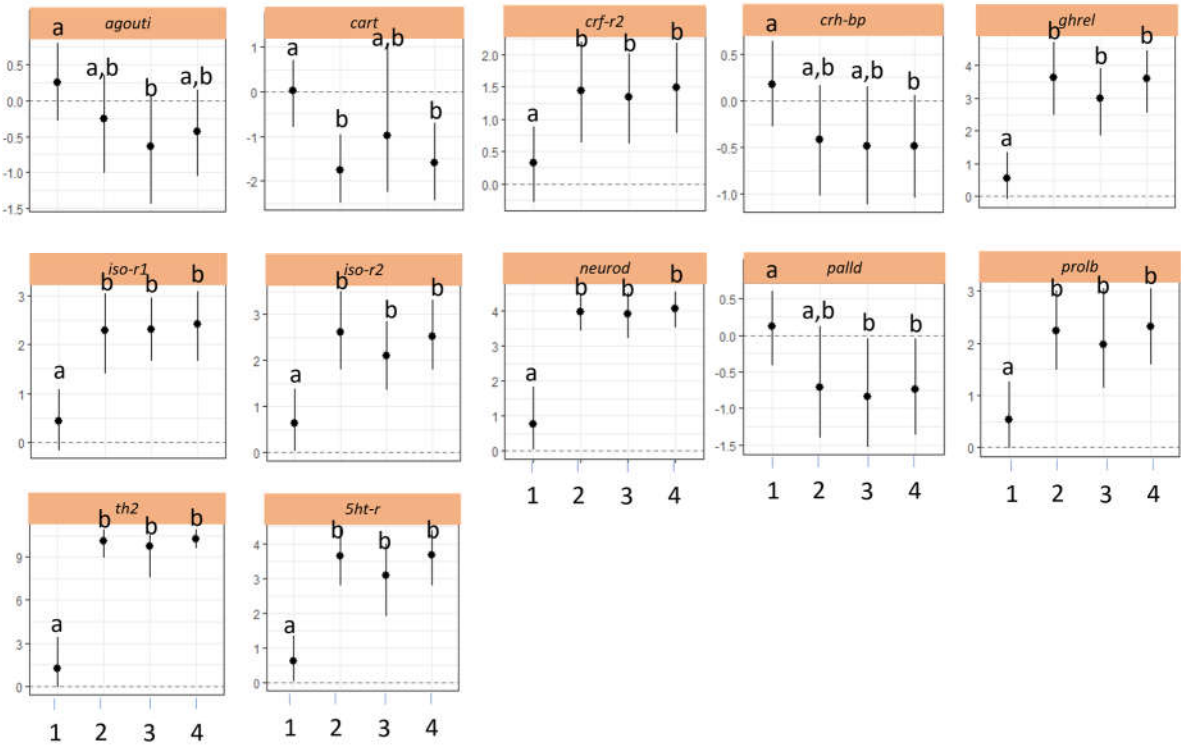

In the optic tectum of males, the expression of *egr-1* in feed-rewarded fish was significantly lower 60 min and 90 min after the treatment compared with remaining treatments (p = 0.044; Fig.S5A), whereas the *erk-1* expression was significantly higher in fish sampled 90 min after the treatment compared with the controls or fish 60 min after treatment ($p \leq 0.016$). The expression of *palld* was significantly lower 90 min after the treatment compared with the controls (p = 0.016). In addition, the expression of *c-fos* was significantly higher 60 min and 90 min after the treatment compared with controls ($p \leq 0.032$). The expression of *citrsyn* was higher at 30 min compared with the remaining treatment ($p \leq 0.024$). The expression of *c-fos* in feed control fish was significantly higher 30 min after the treatment compared with the remaining sampling time points ($p \leq 0.026$; Fig. S5B). The expression of *pyrkin* was higher in fish 30 min after feed control treatment compared with the fish sampled 60 min and 90 min after treatment ($p \leq 0.040$). The expression of IEGs in chased fish was not significantly influenced in the optic tectum (data not shown), but the expression of the metabolic gene *pyrkin* was higher 90 min after chasing compared with the controls (p = 0.044; Fig S5C). The *erk-1* expression in confined fish was significantly higher 60 or 90 min after treatment compared with the controls or the fish sampled 30 min after the treatment ($p \leq 0.016$; Fig. S5D). Moreover, confined fish showed a higher *neurod* expression in all treated fish compared with the controls ($p \leq 0.006$), and in fish sampled 90 min after treatment compared with the fish sampled at 30 min after treatment (p = 0.036). The *citrsyn* expression was lower in fish sampled 30 min after confinement compared with the 60 min group ($p \leq 0.030$). Air-exposed fish showed lower *palld* expression in the optic tectum 60 and 90 min after treatment compared with the controls ($p \leq 0.040$; Fig. S5E). Air-exposed fish, furthermore, showed higher *neurod* expression in the optic tectum at all sampling time points after treatment compared with the controls ($p < 0.001$).

In the optic tectum of males, the expression of *crf1* and *pomc B* in feed-rewarded fish was significantly higher and the expression of *mr* significantly lower 30 min after feeding compared with controls ($p \leq 0.036$; Fig. S5A). The expression of HPI axis-related in feed control and in chased fish was

not significantly influenced by the feed control treatment (data not shown). In confined fish, the expression of *crf-r2* was significantly higher in fish sampled 60 or 90 min after the treatment compared with the controls or fish taken at 30 min after the treatment ($p < 0.012$ Fig. S5D). In addition, confined fish showed a lower *crh-bp* expression in fish sampled 90 min after the treatment compared with the controls ($p = 0.038$). Air-exposed fish showed higher *crf-r2* expression in the optic tectum at all sampling time points after treatment compared with the controls ($p \leq 0.018$; Fig. S5E). In addition, the expression of *crh-bp* in the optic tectum was lower 90 min after air exposure compared with the controls ($p \leq 0.044$).

In the optic tectum of males, the expression of *grp* and *ghrel* was significantly higher and the expression of *cart* was significantly lower 30 min after the feed rewarding compared with the remaining treatments ($p \leq 0.036$; Fig. S5A). The expression of *cck-b* in feed-rewarded fish was significantly higher 90 min after the treatment compared with controls ($p = 0.026$). In the same fish, the expression of *cck-a* was significantly higher in fish sampled 90 min after treatment compared with the controls and fish belonging to the 30 min group ($p \leq 0.034$). In feed control fish, the expression of *cart* was significantly higher and the expression of *agouti* significantly lower 30 min after treatment compared with the controls ($p \leq 0.048$; Fig. S5B). The expression of *ox* in chased fish showed a lower value 30 min after treatment compared with the controls ($p = 0.036$; Fig. S5C). In confined fish, the expression of *grp* was significantly increased in all treated animals compared with the controls, but also higher 60 and 90 min after treatment compared with animals sampled 30 min after treatment ($p \leq 0.016$; Fig. S5D). The *ghrel* expression in confined fish was significantly different between all sampling time points ($p \leq 0.040$). The *ox* expression in confined fish was significantly higher 60 or 90 min after treatment compared with the controls or the fish sampled 30 min after the treatment ($p \leq 0.034$). In addition, the expression of *npy* in the optic tectum of the same fish was only higher 90 min after treatment compared with the controls ($p = 0.042$). In contrast, the expression of *agouti* in the optic tectum was lower 90 min after treatment compared with the controls ($p = 0.012$). Air-exposed fish showed higher *ghrel* expression in the optic tectum at all time points after treatment compared with the controls ($p < 0.001$; Fig. S5E). Air-exposed animals also showed lower *cart* expression 30 and 90 min after treatment compared with the controls ($p < 0.008$), and lower *agouti* expression 60 min after treatment compared with the controls ($p = 0.034$).

In the optic tectum of males, the expression of *prola* in feed-rewarded fish was significantly higher 30 min after the treatment compared with controls and fish 90 min after treatment ($p \leq 0.048$; Fig. S5A). Similarly, the expression of *5ht-r* was significantly higher 30 min and 60 min after the treatment compared with controls ($p \leq 0.002$). The expression of *iso pre, dopar 2a, opio d1a* and *prolb* was higher and the expression of *mtor* significantly lower at 30 min compared with the remaining treatment ($p \leq 0.024$). The expression of *tph* was higher in fish 30 min after treatment compared with the controls ($p = 0.022$). The expression of *prolb* was lower in fish 30 min after feed control treatment compared with the fish sampled 60 min and 90 min after treatment ($p \leq 0.040$; Fig. S5B). The expression of *iso-r1* in chased fish showed a higher value 60 min after treatment compared with the controls ($p = 0.046$; Fig. S5C). In confined fish, the expression of *prola* was significantly lower in fish sampled 60 min after the treatment compared with the controls ($p = 0.038$; Fig. S5D), but the expression of *tph* higher in the same fish ($p = 0.024$). Moreover, confined fish showed a higher *5ht-r, th2* and *prolb* expression in all treated fish compared with the controls ($p = 0.028$), and in fish sampled 60 and 90 min after treatment compared with the fish sampled at 30 min after treatment ($p = 0.044$). The expression of *dopar 2b* in the same fish was also found to be lower in fish 30 min after the treatment compared with the controls ($p = 0.048$). Moreover, the expression of *mtor* was higher in confined fish sampled 60 and 90 min after treatment compared with the controls, and the expression of *dopar 2a* was higher in fish sampled 60 and 90 min after treatment compared with the controls and fish taken 30 min after the treatment ($p \leq 0.030$). The expression of *pyrkin* was lower 30 min and 90 min after treatment compared with the controls ($p \leq 0.012$). The expression of *opio d1b* was lower in confined fish sampled 90 min after treatment compared

with the controls ($p = 0.028$). In contrast, the expression of *opio d1a* was higher in confined fish sampled 60 min after treatment compared with the controls ($p = 0.030$). Finally, the expression of *iso-r1* in chased fish was different at all timepoints analysed in this study ($p = 0.028$). Air-exposed fish showed higher *5ht-r, th2,* both isotocin receptors expression and lower *prolb* expression in the optic tectum at all sampling timepoints after treatment compared with the controls ($p \leq 0.008$; Fig. S5E).

Figure S4. Gene expression profiles (expressed as marginal means derived from the Bayes models performed on the log2 values of the normalized gene expression values) in the rhombencephalon of male control fish (group 1) and fish after the treatment (30 min = 2; 60 min = 3; 90 min = 4) whereby the following different treatments have been applied as described in the Material & Methods section: feed rewarding, feed control, chasing, confinement and air exposure, marginal means ± SEM; n = 6 per treatment, means of groups with the same letters are not significantly different from each other, $p < 0.05$).

6A : Feed reward

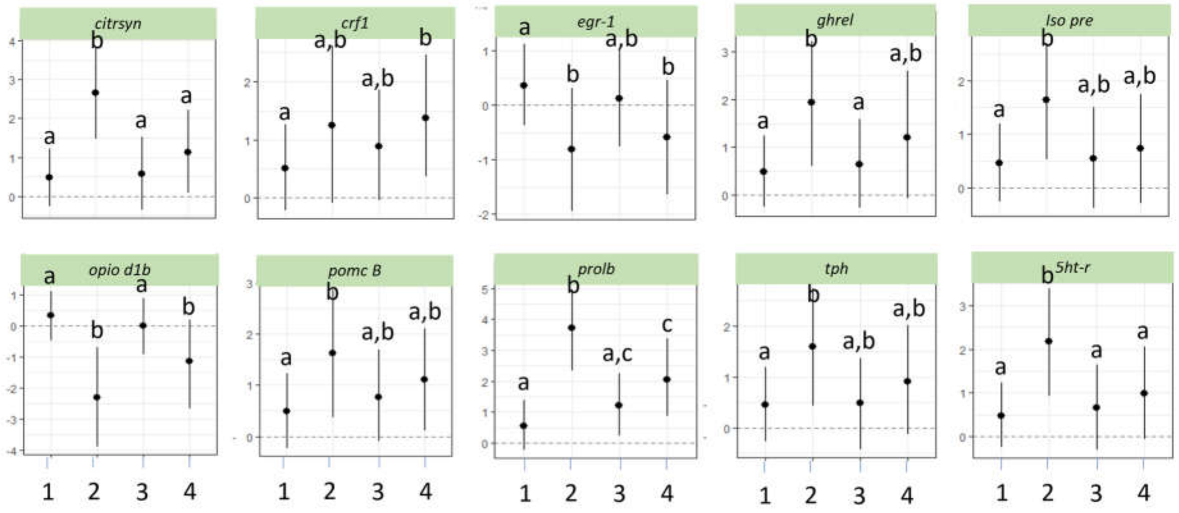

6B : Feed Contr

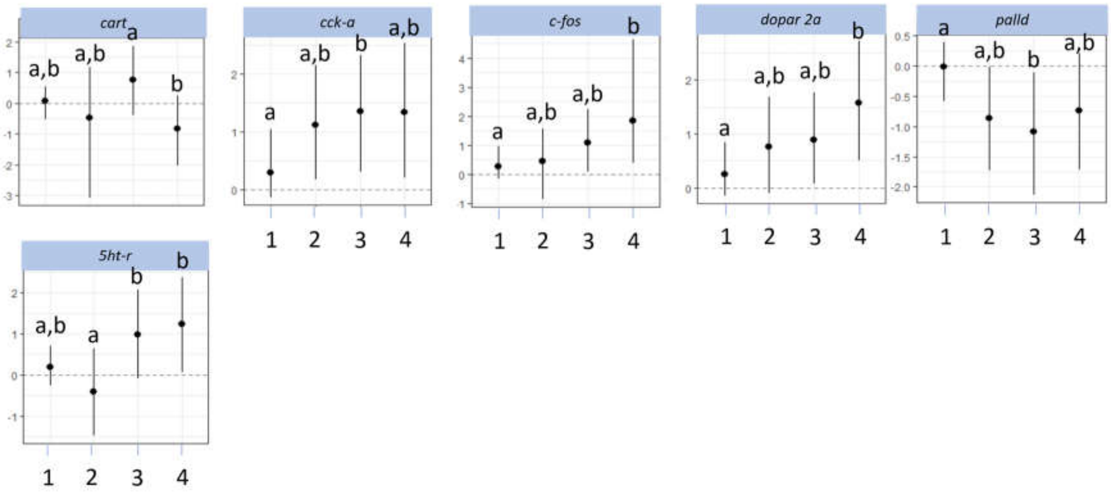

6C : Chasing

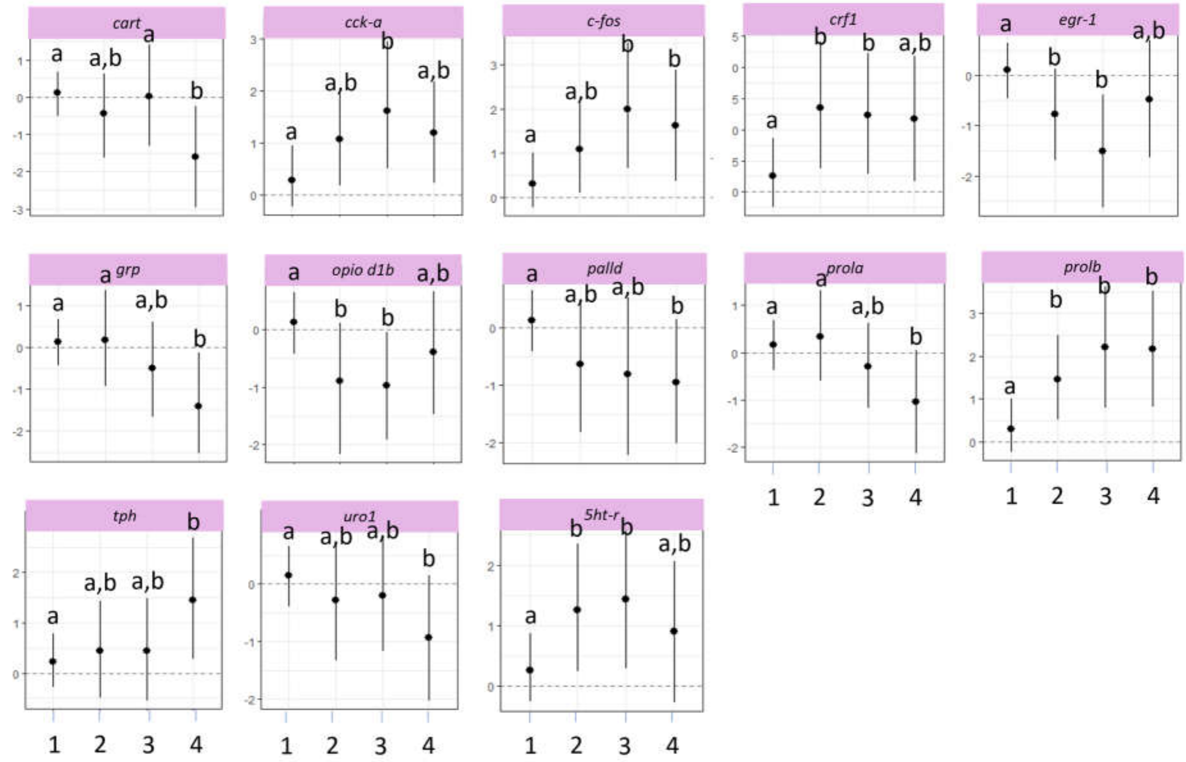

6D : Confinement

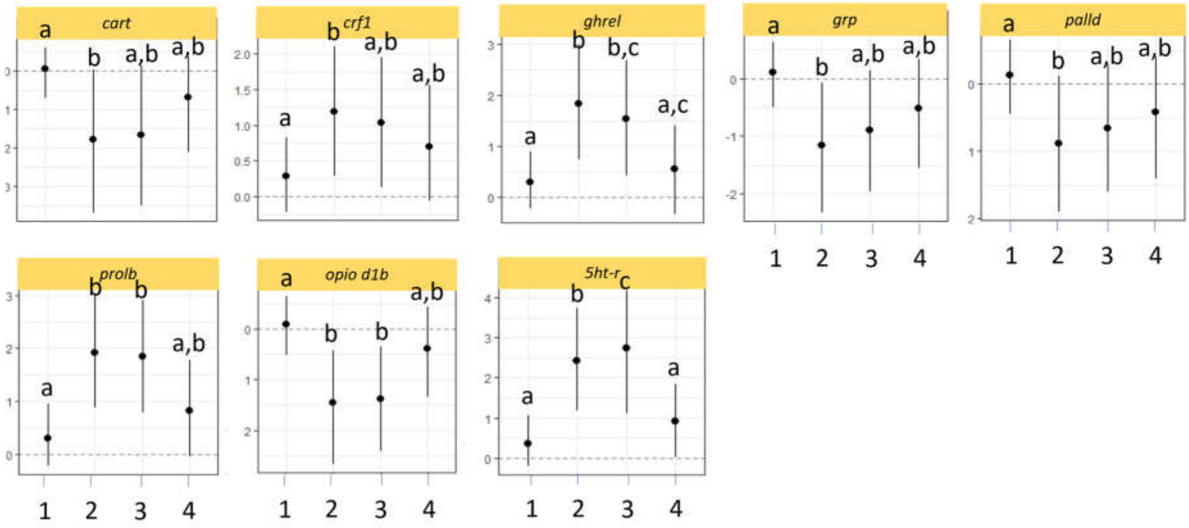

6E : Air

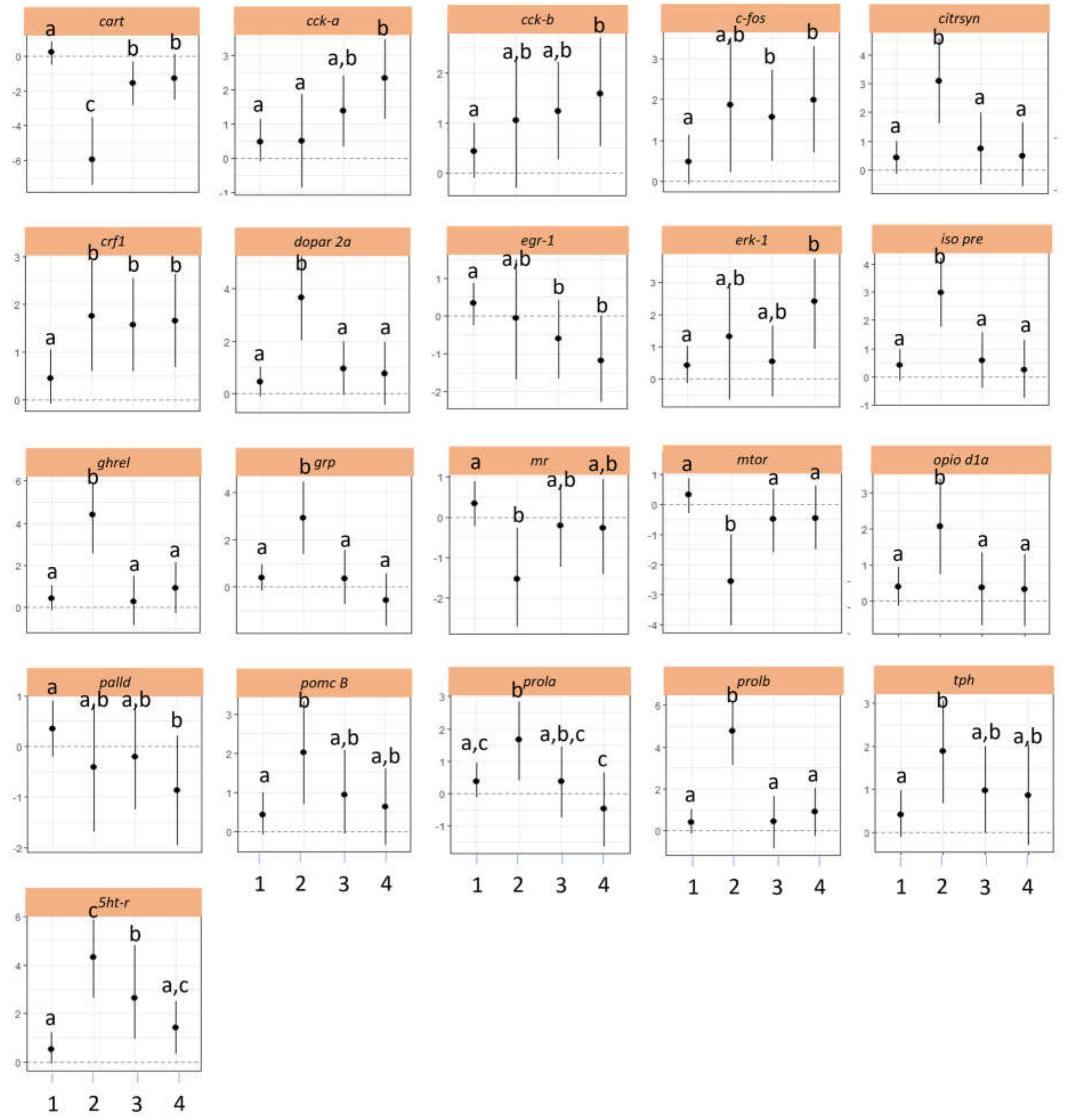

In the rhombencephalon of males, feed rewarding resulted in lower *egr-1* expression in fish 30 min and 90 min after treatment compared with the controls ($p < 0.020$; Fig. S6A). In addition, the expression of *citrsyn* in feed-rewarded fish was significantly higher in fish sampled 30 min after treatment compared with the remaining fish groups ($p \leq 0.048$). Feed control fish showed higher *c-fos* expression 90 min after treatment compared with the controls ($p \leq 0.030$; Fig. S6B). The same fish also showed lower expression of *palld* in the rhombencephalon of fish 60 min after treatment compared with the controls ($p = 0.038$). In chased animals, the expression of *egr-1* in the rhombencephalon was lower in fish 30 and 60 min after treatment compared with the controls ($p \leq 0.016$; Fig. S6C). In chased animals, the expression of *c-fos* in the rhombencephalon was higher in fish 60 min and 90 min after treatment compared with the controls ($p \leq 0.032$), and lower *palld* expression after 90 min compared with the controls ($p = 0.034$). Confined fish showed a lower *palld* expression in fish 30 min after treatment compared with the controls ($p = 0.038$; Fig. S6D). Air-exposed fish showed a lower *egr-1* expression and a higher *c-fos* expression in fish 60 and 90 min after treatment compared with the controls ($p \leq 0.046$;

Fig. S6E), whereas *erk-1* showed a higher and *palld* a lower expression 90 min after treatment compared with the controls ($p \leq 0.016$).

In the rhombencephalon of males, the expression of *crf1* in feed-rewarded fish was significantly higher in all treated fish compared with the controls ($p \leq 0.032$; Fig. S6A). In addition, the *pomc B* expression was lower 30 min after feed rewarding compared with the controls ($p = 0.042$). The feed control fish showed no differences in gene expression (data not shown). In chased animals, the expression of *crf1* in the rhombencephalon was higher in fish 30 min and 60 min after the treatment compared with the controls ($p \leq 0.042$). In the same animals, a lower expression of *uro 1* in the rhombencephalon of fish 90 min after treatment compared controls was observed ($p = 0.028$). Confined fish showed a higher *crf1* and *pomc B* expression in fish sampled after 30 min compared with the controls ($p = 0.030$; Fig. S6D). Air-exposed fish showed a higher *crf1* expression in all treated fish compared with the controls ($p < 0.034$; Fig. S6E), and a lower *mr* expression 30 min after treatment compared with the controls ($p = 0.008$).

In the rhombencephalon of males, the expression of *ghrel* in feed-rewarded fish was significantly higher at 30 min after the treatment compared with controls and at 60 min after the treatment ($p \leq 0.040$; Fig. S6A). The feed control fish also showed lower expression of *cart* in the rhombencephalon at 90 min after treatment compared with the 60 min group ($p = 0.028$; Fig. S6B). The same fish showed lower expression of *cck-a* in the fish 60 min after treatment compared with the controls ($p = 0.046$). In chased animals, the expression of *grp* in the rhombencephalon was reduced in fish 90 min after the treatment compared with the controls and fish samples 30 min after treatment ($p \leq 0.028$; Fig. S6C). Moreover, chased animals showed a lower expression of *cart* in the rhombencephalon in fish sampled after 90 min compared with the controls and fish of the 60 min group ($p \leq 0.044$). The same fish showed higher expression of *cck-a* in the fish 60 min after treatment compared with the controls ($p = 0.012$). Confined fish showed a lower *grp* and *cart* expression in fish sampled 30 min after treatment compared with the controls ($p \leq 0.034$; Fig. S6D). The confined fish also showed higher *ghrel* expression in fish 30 min and 60 min after treatment compared with the controls ($p \leq 0.026$). Air-exposed fish showed a higher *grp* and *ghrel* expression in fish 30 min after treatment compared with the remaining treatments ($p < 0.004$; Fig. S6E). In the same fish, a lower *cart* expression in all air-exposed fish was observed compared with the controls ($p \leq 0.032$). In addition, these fish showed a higher *cck-a* and *cck-b* expression at 90 min after treatment compared with the controls ($p = 0.022$), whereby the difference between the mRNA expression levels of *cck-a* were also different between 30 min after treatment and 90 min after treatment ($p = 0.034$).

In the rhombencephalon of males, the expression of *5ht-r* in feed-rewarded fish was significantly higher in fish sampled 30 min after treatment compared with the remaining fish groups ($p \leq 0.048$; Fig. S6A). In the same fish, the expression of *iso pre* was higher in fish sampled 30 min after treatment compared with the controls ($p = 0.014$). The expression of *prolb* was significantly higher in fish sampled after 30 min after treatment compared with the controls and fish belonging to the 90 min groups ($p \leq 0.028$). Moreover, feed rewarding resulted in higher expression of *tph* in the rhombencephalon in fish sampled 30 min after treatment compared with the controls ($p < 0.030$). The expression of *opio d1b* was lower in fish sampled 30 min and 90 min after treatment compared with the remaining two groups ($p \leq 0.040$). The feed control fish showed lower expression of *5ht-r* in the rhombencephalon of fish sampled 30 min after treatment compared with the 60 min and the 90 min groups ($p < 0.048$; Fig. S6B). In chased animals, a lower expression of *prola* was observed in the rhombencephalon in fish of the 90 min group compared with the controls and fish sampled 30 min after treatment ($p \leq 0.030$; Fig. S6C), whereas the expression of *prolb* was different in all treatments compared with the controls ($p \leq 0.018$). In the same animals, the expression of the *5ht-r* was higher fish sampled 30 min and 60 min after treatment compared with the controls ($p \leq 0.030$). The expression of the *tph* was higher in fish sampled 90 min

after treatment compared with the controls ($p \leq 0.028$). In addition, chased fish showed a lower *opio d1b* expression in fish sampled 30 and 60 min after treatment compared with the controls ($p \leq 0.030$). Confined fish showed a higher *5ht-r* expression in fish of the 30 min and 60 min group compared with the other two groups ($p \leq 0.022$; Fig. S6D). These fish also showed a higher *prolb* expression and a lower expression of *opio d1b* in fish 30 and 60 min after treatment compared with the controls ($p \leq 0.004$). Air-exposed fish showed a higher *prola* expression in fish of the 30 min group compared with the controls and fish sampled 90 min after treatment ($p \leq 0.046$; Fig. S6E). The expression of *5ht-r* and *tph* was also higher between fish sampled 30 min and 60 min after treatment and the controls ($p \leq 0.004$). In addition, the expression of *iso pre, citrsyn, dopar 2a, opio d1a* and *prolb* was significantly higher and the expression of *mtor* significantly lower at 30 min after treatment compared the remaining treatment ($p \leq 0.028$).
